# Transient pain and discomfort when wearing high-heeled shoes

**DOI:** 10.1038/s41598-024-59966-9

**Published:** 2024-04-23

**Authors:** Hour Matar Abdulla Almadhaani, Ravindra S. Goonetilleke, Albert Wijeweera, Raja Jayaraman, Luximon Ameersing, Ahsan H. Khandoker, S. B. Mohd. Tamrin

**Affiliations:** 1 Department of Management Science and Engineering, Khalifa University, Abu Dhabi, UAE; 2 Healthcare Engineering Innovation Center, Khalifa University, Abu Dhabi, UAE; 3 Industrial Design, Georgia Tech Shenzhen Institute/Tianjin University (GTSI), Shenzhen, China; 4 Department of Biomedical Engineering, Khalifa University, Abu Dhabi, UAE; 5 Department of Environmental and Occupational Health, University of Putra, Serdang, Malaysia

**Keywords:** Posture, High heels, Comfort, Pain level, Footbed, Foot pressure, Psychology, Health care, Medical research, Risk factors, Engineering

## Abstract

In the dynamic world of fashion, high-heeled footwear is revered as a symbol of style, luxury and sophistication. Yet, beneath the facade of elegance of classy footwear lies the harsh reality of discomfort and pain. Thus, this study aims to investigate the influence of wearing high-heeled shoes on the sensation of pain across different body regions over a period of 6 h. It involved fifty female participants, all habitual wearers of high-heeled shoes, aged between 20 and 30 years. Each participant kept a record of their perceptions of pain and discomfort every hour for a total of 6 h using a 0–10 pain scale with 0 indicating no pain and 10 indicating severe pain. The findings reveal a progressive rise in pain throughout wear, with the most intense pain reported in the back, calcaneus, and metatarsals. The analysis shows that after approximately 3.5 h, participants experience significant increases in pain levels. However, the relationship between heel height and pain is not linear. It appears that a heel height of 7.5 cm is the threshold where overall body pain becomes significant. The study suggests that a duration of 3.5 h of wear and a heel height of 7.5 cm serve as critical points to decrease overall body pain. Moreover, beyond this heel height, knee pain diminishes compared to other body areas possibly due to the shift towards a more neutral posture. The study findings, coupled with the recommendations, can assist footwear designers in crafting not only stylish but also comfortable shoes.

## Introduction

As fashion continues to evolve, high-heeled shoes are emerging as a luxurious item, gaining popularity among women^1–4^. Such footwear have been associated with muscle overuse, leading to muscular discomfort and occasional injuries, despite some claims suggesting no significant change or even a decrease in muscle activity^5–12^. The higher the heel, the more notable the alteration in the ankle–foot complex, setting off a chain reaction that affects the entire body, extending from the foot–ankle, to the knee and even to the spine^10,13–16^.

A key contributor to this insufficiently explored chain reaction is the footbed. Injuries and health issues often stem from inadequately designed footbeds, primarily because they may fail to offer comprehensive foot support or provide the necessary support in the right areas^17^. This reduced support results in heightened foot pressure, particularly on the metatarsal heads, leading to changes in the center of pressure (COP)^18–26^. Moreover, the task of maintaining postural balance becomes increasingly difficult when donning poorly designed high-heeled shoes, elevating the potential for accidents and falls^6,27–29^. Prolonged use of high heels has the potential to shift the center of mass (COM), which could result in increased stress and instability within the foot–ankle complex^30,31^ thereby increasing midfoot pain and even changing the arch morphology^25,32^.

Consequently, individuals who regularly wear high heels tend to employ a conservative strategy to alter their centre of mass in relation to the centre of pressure^33,34^. These adaptive strategies encompass a spectrum of techniques, ranging from structural adjustments such as trunk and pelvis rotations^10,33,35,36^ to modifications in muscle–tendon architecture^36–38^ leading the body away from its natural or neutral posture^39^. Approximately 58% of individuals who regularly wear high heels experience low back pain (LBP) believed to be associated with an increased lumbar lordosis, possibly due to pelvic rotations^40–42^. However, some studies have found either a decreased lordosis (or kyphosis) with increasing heel heights^43^ or no significant impact on lumbar lordosis^44,45^.

Even though controversial, Baaklini et al.^43^, claimed that wearing heels with a height of 4–7 cm does not have a detrimental effect on comfort, balance, or mobility. Hapsari and Xiong^27^ have also shown that functional mobility is affected at a height of 7 cm. Similarly, Lee et al.^41^ found that 55% of respondents surveyed had issues with a heel height of 6–9 cm. However, Baaklini et al.^43^ cautioned that further investigation is needed to understand the effects over time and to determine the point at which heel height becomes biomechanically disadvantageous. Moreover, while studies have investigated the consequences of prolonged high-heel usage, highlighting issues such as heel, forefoot, midfoot, back, and calf pain^32,46,47^, there appears to be a dearth of literature addressing the day-long discomfort or pain experienced when wearing high-heel shoes, to the best of the authors' knowledge.

Therefore, it is imperative to investigate the impact of high-heeled shoes during daily use when wearing shoes with different heel heights and identifying the specific areas that are affected to prevent any undesirable long-term consequences. The primary goal being to find the threshold of the wearing duration of high-heeled shoes and the threshold of heel height to minimize pain and discomfort.

## Method

The study was performed in two phases. In the first, we focused on crafting a pain questionnaire, while the second phase involved a cross-sectional study. In the second, data was recorded at specified intervals, without any researcher intervention or manipulation. Despite its comparatively lower degree of control compared to formal experiments, this approach has several merits such as real-world relevance, fidelity to authentic scenarios, diverse participant and activity representation, and overall practicality.

### Pain questionnaire design

One of the co-authors (HMAA) conducted a preliminary study involving five different high-heeled shoes of varying heights, each worn for 5 h on separate days. The objective was to understand the emergence of discomfort or pain over time. Extended usage of high heels may result in compensatory postural adjustments, resulting in increased strain on the upper body due to altered spinal curvature and balance mechanisms. Thus, the potential discomfort on the whole body was assessed. Hourly observations were made, and the level of comfort or discomfort was documented using a scale similar to the Body Part Discomfort Scale^48^, accompanied by pictographs to gauge the pain/discomfort level. A selection of the preliminary study results is presented in Appendix A. These findings indicated that discomfort or pain gradually intensifies over time. Consequently, based on the insights gained from this pilot study, a pain/discomfort survey was developed as shown in Appendix B.

The study was approved by the Khalifa University ethics review board (Protocol #: H22-044). All methods and materials were carried out under strict guidelines and regulations provided by the institutional ethics review committee. The data collected were anonymous and strict confidentiality was maintained by recording the data using numbers instead of names.

### Participants

Fifty female participants aged 20–30 years were instructed to record their experiences when wearing high-heeled shoes, detailing the areas and the severity of pain during the period March–April 2023. Each participant provided written informed consent to participate in the study. The demographic characteristics of the study participants are presented in Table 1. To mitigate the influence of pre-existing conditions, the study exclusively enrolled females who did not have any foot disorders or abnormalities, postural instability, spinal or knee issues, or a history of surgeries. Foot anatomy, anthropometry or fitness levels were not assessed.

**Table 1:** Descriptive statistics of participants (n = 50).

	Mean	SD	Max	Min
Age (years)	24.98	2.15	29	21
Weight (Kg)	62.7	9.98	85	45
Height (cm)	162.92	5.16	177	154
Foot Size (cm)	38.37	1.15	41	36
BMI	24.56	1.53	27.4	20.7

### Procedure

**Figure 1:**
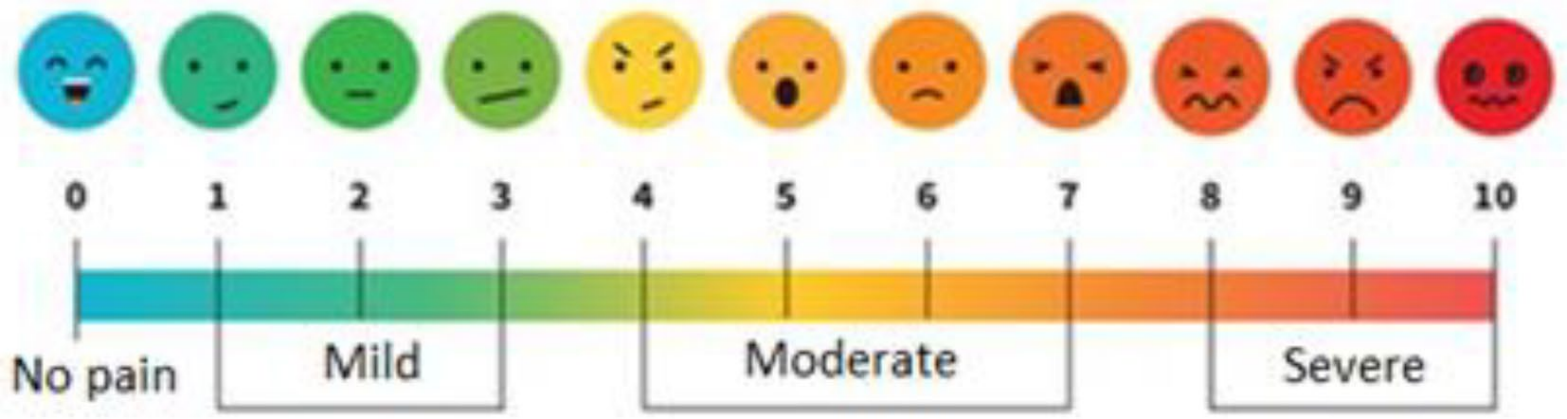
Pain measurement scale used to determine pain level^49^.

The evaluations were sought to determine whether pain levels increased across the entire body as the duration of shoe-wearing extended. To enhance the clarity of the survey process for the participants, the researcher showed an example where she donned high-heeled shoes with a height of 11.2 cm (Appendix C). A modified version of the pain assessment scale^49^ (Fig. 1) was used to assess the extent of discomfort/pain experienced across various body regions during a 6-h duration. The form helped illustrate the experience using visual depictions of the pain locations, accompanied by pain intensity ratings and descriptions of the experimenter.

Each participant was tasked with wearing their uncomfortable high-heeled shoes for 6 h, adhering to the steps as demonstrated and explained by the researcher in the self-conducted survey (Appendix C). Throughout the 6-h duration, all participants performed their typical activities including sitting, standing, and walking on diverse surfaces. Such a procedure was intentional so that there is a realistic representation of daily life, avoided artificial constraints, has statistical randomness and enhanced generalizability. Subsequently, each study participant filled out the form provided to them (Appendix B), offering feedback on their individual experiences when wearing high-heeled shoes.

## Results

**Figure 2:**
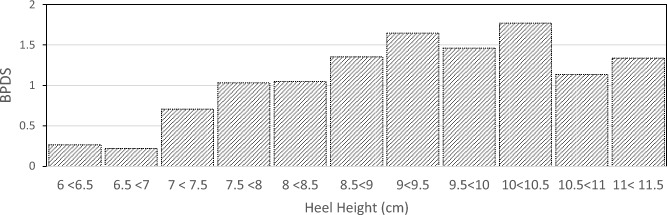
Mean level of pain across all body regions (BPDS) with increasing heel height on all body regions.

The participant responses were summarized with descriptive statistics. The heel height worn by participants varied from 6.4 to 11.3 cm. To avoid single-point data, the heel heights were grouped into 0.5 cm intervals. Figure 2 displays the body part discomfort severity (BPDS)^50^ across the entire body for different heel heights. The BPDS was calculated as follows:

$$BPDS = \frac{{\mathop \sum \nolimits_{body \,parts = 1}^{N} Pain}}{Number\, of\, nonzero \,values }$$

Figure 2 shows that there is little to no pain (scale value of less than 1), up to a heel height of approximately 7.5 cm. Beyond this point, there is a gradual increase in reported pain with increasing heel height. Given this trend, further analysis was performed examining participants wearing heel heights below 7.5 cm and those at or above 7.5 cm.

**Figure 3:**
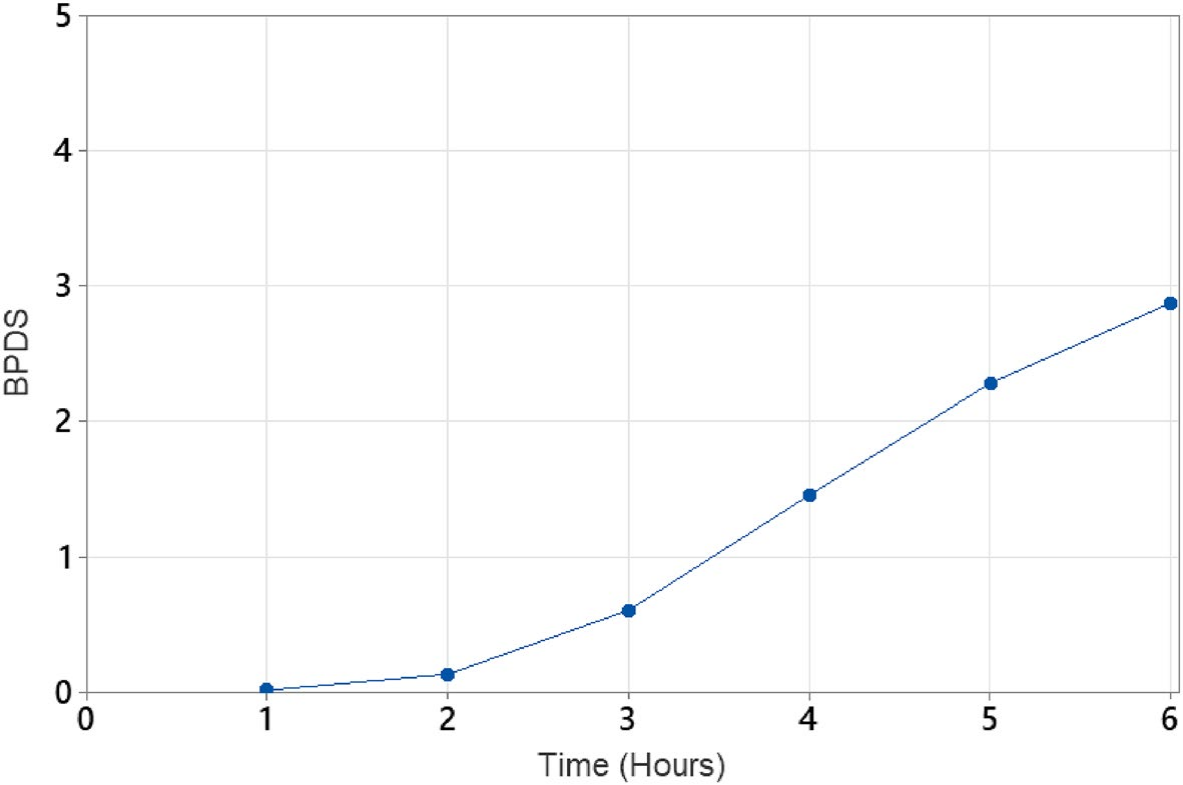
Mean level of pain across whole body (BPDS) over time.

**Figure 4:**
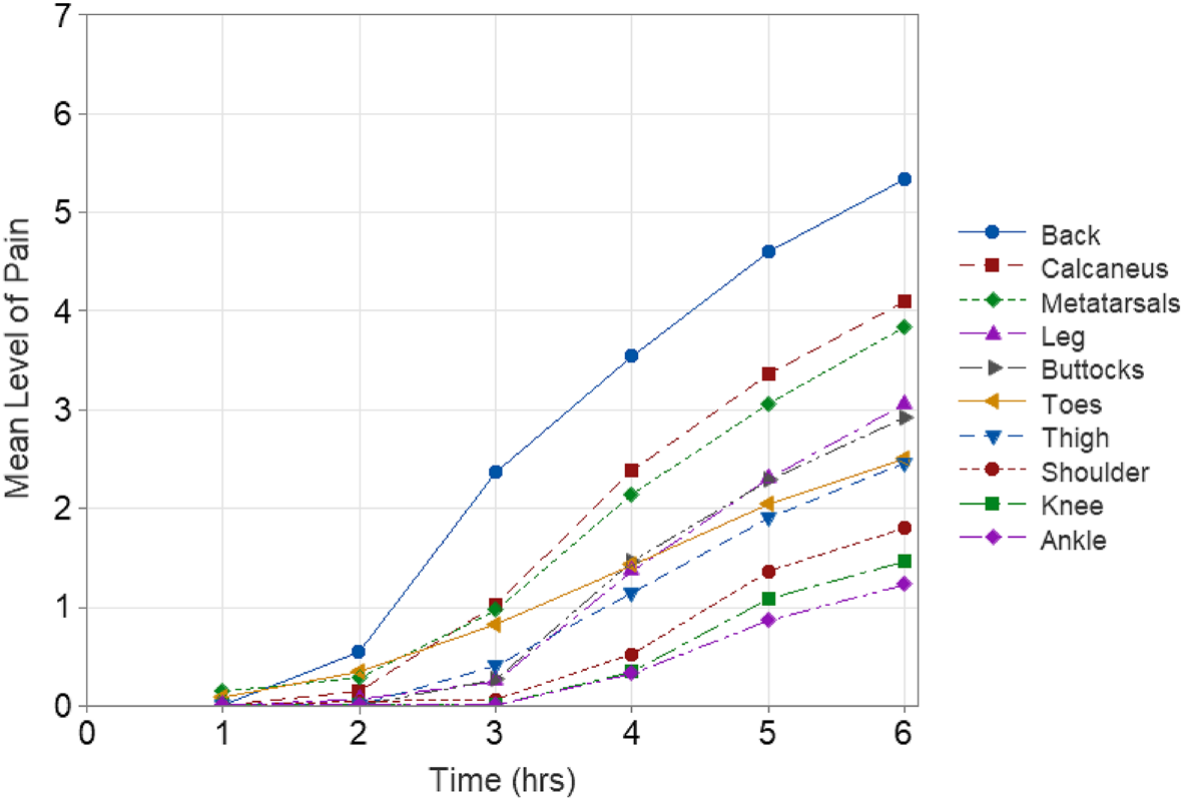
Mean level of pain over time on various body parts.

**Figure 5:**
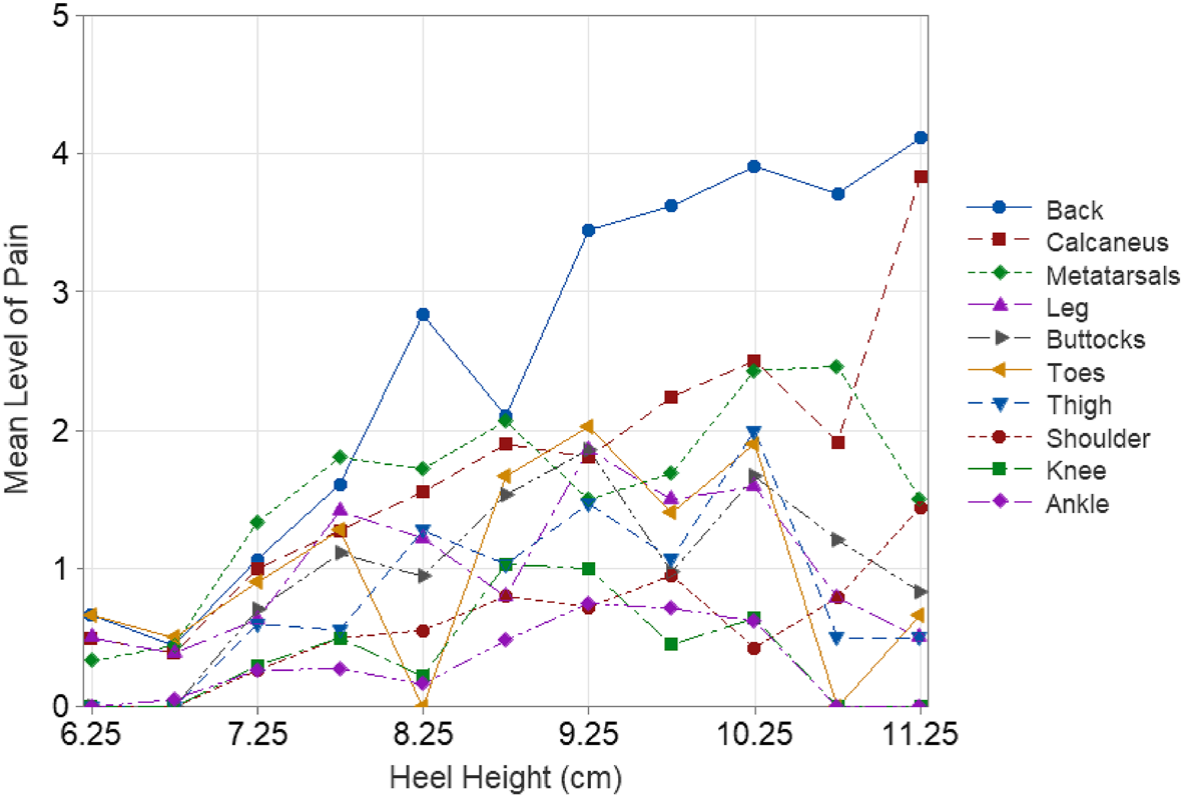
Mean level of pain of the various body parts when wearing differing heel heights of shoes.

In relation to the duration of wearing high-heeled shoes, mild discomfort becomes noticeable (at or above level 1) after approximately 3.5 h of wear when considering all the different body parts as a whole (i.e., mean of all ratings) (Fig. 3). The variation in the severity of pain over time in the various body parts is shown in Fig. 4 while the variation over heel height is shown in Fig. 5. The heel heights shown are the mid-point of the corresponding range of heel height. For example, the category linked with 6–6.5 cm is presented as 6.25 cm, aiming to streamline and enhance the clarity for identifying patterns, particularly in the context of using a bar chart. Figure 4 illustrates that back pain starts to develop after wearing high-heels for about 2.25 h while the pain in other parts of the body starts after around 3 h.

Due to the consistent pain patterns observed in the different parts of the body (Figs. 4 and 5), a correlation analysis (Table 2) was performed. The correlations reveal a consistent trend: pain levels increase across all areas of the body over time. However, there seems to be no such observable trend concerning heel height. The total body pain remained below 1 (indicating an absence of pain) for heel heights under 7.5 cm (Fig. 2). In light of this finding, a multiple regression analysis was done, using heel height and duration of wear as predictive factors. This analysis was performed separately for heel heights below 7.5 cm and those at or above 7.5 cm. This division was made considering the apparent threshold at 7.5 cm, beyond which pain becomes noticeable (Fig. 2).

**Table 2:** Correlation coefficients between heel height, time and pain level.

	Height	Time	Shoulder	Back	Butt	Thigh	Knee	Ankle	Leg	Mets	Calc	Toes
Time	0											
Shoulder	0.24	**0.64**										
Back	0.43	*0.74*	*0.66*									
Buttocks	0.17	**0.64**	**0.53**	**0.62**								
Thigh	0.18	**0.56**	0.38	**0.61**	*0.72*							
Knee	0.02	0.49	0.50	0.44	**0.58**	**0.54**						
Ankle	0.08	0.47	0.38	0.45	**0.55**	**0.57**	**0.71**					
Leg	0.08	**0.62**	0.42	**0.60**	*0.67*	**0.61**	0.46	**0.58**				
Metatarsals	0.18	*0.65*	0.49	**0.62**	*0.77*	*0.65*	**0.55**	0.50	*0.66*			
Calcaneus	0.35	*0.75*	**0.61**	*0.74*	**0.59**	**0.57**	0.37	0.39	0.47	**0.56**		
Toes	0.05	0.42	0.23	0.37	0.50	**0.56**	**0.57**	**0.54**	0.42	0.44	0.29	
Total	0.26	*0.80*	0.66	*0.83*	*0.86*	*0.82*	*0.70*	*0.70*	*0.78*	*0.83*	*0.76*	**0.64**

Coefficients 0.65 or greater are in italics. Coefficients 0.5 or greater are in bold.

The corresponding models and their coefficients are given in Table 3. The *p*-values for the related Time of wearing coefficients are less than 0.05 for all body parts. The regression coefficients of heel height show statistical significance at a 1% level for all body parts, except the ankle, toes, and leg. This suggests that heel height influences the pain levels in the shoulder, back, thigh, knee, metatarsals, and calcaneus. The findings also indicate that all coefficients express the hypothesized positive indications for heel height and duration of wear across all examined body regions when the heel height is under 7.5 cm. This suggests that an increase in these factors correlates with higher reported pain levels in the specified body areas. With regression coefficients of 0.76, for example, maintaining other variables constant, an increase of 1.3 cm in heel height is projected to correspond to a 1-point rise in back pain on a scale from 1 to 10. Similarly, each additional 2-h interval of wearing high-heeled shoes tends to raise the level of back pain by approximately 1-point as the regression coefficient is 0.5.

**Table 3:** Regression coefficients of Body Pain on Heel Height and duration of use.

	Shoulder	Back	Buttocks	Thigh	Knee	Ankle	Leg	Mets	Calcaneus	Toes
Const	− 3.14	− 6.21	− 9.08	− 6.97	− 3.34	− 2.46	− 2.98	− 11.94	− 7.44	− 2.92
	(− 1.91)	(− 8.01)	(− 1.50)	(− 1.87)	(0.45)	(− 0.45)	(− 0.35)	(− 2.28)	(− 6.02)	(0.08)
Heel Height	0.42	0.76	1.25	0.97	0.45	*0.32*	*0.32*	1.57	0.94	*0.31*
	(0.12)	(0.71)	(*0.03*)	(*0.09*)	(− 0.12)	*(*− *0.01)*	*(*− *0.09*)	(*0.13*)	(0.49)	*(*− *0.07*)
Time	0.11	0.50	0.24	0.16	0.12	0.12	0.37	0.56	0.48	0.44
	(0.44)	(1.29)	(0.74)	(0.62)	(0.35)	(0.29)	(0.72)	(0.85)	(0.99)	(0.52)
R-sq	0.33	0.51	0.35	0.20	0.17	0.22	0.40	0.52	0.60	0.30
	(0.49)	(0.75)	(0.49)	(0.39)	(0.29)	(0.25)	(0.41)	(0.45)	(0.70)	(0.17)
*p*-value	0.00	0.00	0.00	0.00	0.01	0.00	0.00	0.00	0.00	0.00
	(0.00)	(0.00)	(0.00)	(0.00)	(0.00)	(0.00)	(0.00)	(0.00)	(0.00)	(0.00)

The top line corresponds to a heel height below 7.5 cm. The values for heel heights 7.5 cm or above are in parenthesis. The regression coefficients in italics are those having *p*-values above 0.05.

For heel heights below 7.5 cm, the heel height coefficients have *p*-values that are less than 0.05 for all body parts except for the ankle, leg and toes, where the relevant *p*-values are 0.09, 0.33, and 0.53 respectively. For 7.5 cm or above heel heights, the p-values are less than 0.05 for all body parts except for the ankle, leg, toes, metatarsals, thigh and buttocks where the *p*-values are 0.82, 0.29, 0.58, 0.18, 0.26 and 0.74 respectively (Shown in italics in Table 3). Table 3 also includes R^2^ values to indicate the goodness-of-fit for each regression model. The models for calcaneus pain (below 7.5 cm) and for back (at or above 7.5 cm) have the highest R^2^ (0.60 and 0.75 respectively).

**Figure 6:**
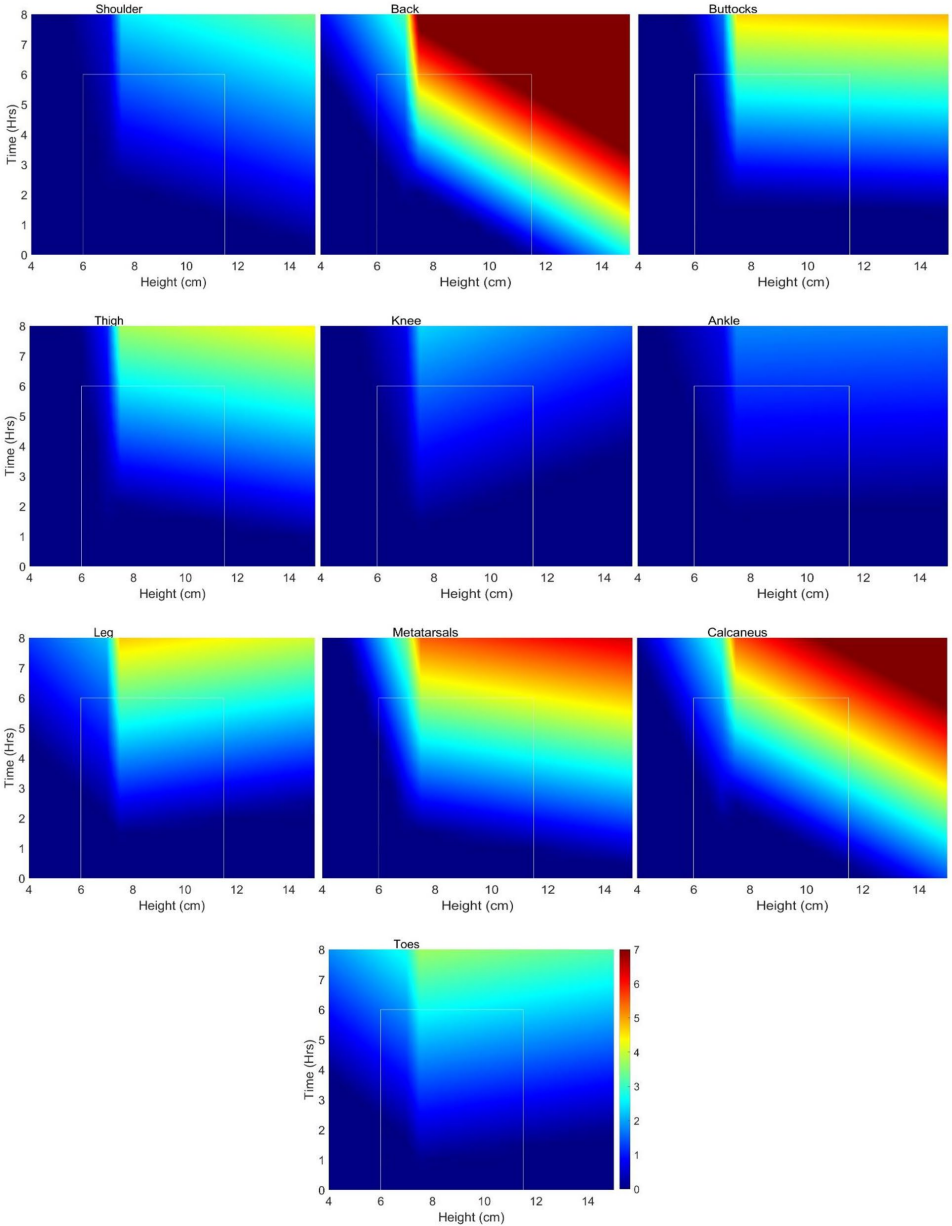
Pain heat maps for different body parts. The color range is up to a pain level of 7 to indicate severe pain. All predicted negative pain values have been set to zero. The light-white rectangle within each plot indicates the region tested in experiment.

Based on the regression equation from Table 3, the predicted pain is plotted for a time duration of 0–8 h and a heel height of 6–15 cm (Fig. 6). In Fig. 6, the pain heat map color range is up to a pain level of 7 to indicate severe pain. The white rectangle within each plot indicates the experimental data region. We have shown the color plot beyond the regions tested to give a better overall picture of the pain realms.

## Discussion

The pain levels experienced by 50 participants wearing high-heeled shoes for 6-h was evaluated. They assessed their pain on the shoulder, back, pelvis, thigh, knee, leg, ankle, toes, metatarsals, and calcaneus using a pain scale ranging from 0 to 10. Consistent with existing literature^10,13–15^, it is seen that the higher the heel, the greater the pain experienced when wearing high-heeled shoes. The pain experienced by subjects appear to be due to two main causes: excessive pressure and muscle fatigue. The bony prominence pain is likely due to excessive pressure^16^ and other location pain is most likely due to muscle fatigue. The back, metatarsals, and calcaneus experience the most amount of pain when wearing high-heeled shoes. Extended use of high heel shoes is also known to increase midfoot pain^25,32^. The reduced support, as a result of poor footbed designs, results in heightened foot pressure, particularly on the metatarsal heads, leading to changes in the center of pressure (COP)^18–26^.

Correlation and regression analysis show that there is a significant relationship between heel height, duration of wear, and subjective pain levels in various body parts among high-heel shoe wearers. Also, heel heights smaller than 7.5 cm seem to be the necessary heel height to avoid pain. Our findings do support Baaklini et al.^43^ who claimed 4–7 cm heel heights do not affect comfort. Our data shows that pain is below level 1 up to heel heights of 7.5 cm. Mild pain (at or above a level of 1 on the pain scale) starts at around 7.5 cm of heel height. Our findings are in partial agreement with Lee et al.^41^ asserting that 55% of people they surveyed had issues with a heel height of 6–9 cm. We hypothesize that the remaining 45% who do not experience any pain could be in the heel height range of 6–7.5 cm. Pain levels keep increasing as heel height increases, the reduction in pain level beyond 10.5 cm is not statistically significant (*p* > 0.05). The reduction could be due to a small sample size or another possibility is that those high heels are well designed to conform to body dynamics through a better match of COM and COP, which can be judged by an expert high-heel wearer at time of purchase. One important result based on this research is the change in sign of knee pain from positive to negative below 7.5 cm and above 7.5 cm heel height (Table 3). For the low heel heights knee pain increases with heel height but then switches sign beyond 7.5 cm. This is because, at higher heel heights, the knee is likely in a fully extended or neutral posture reducing any pain or discomfort to account for balance through postural adaptations.

**Figure 7:**
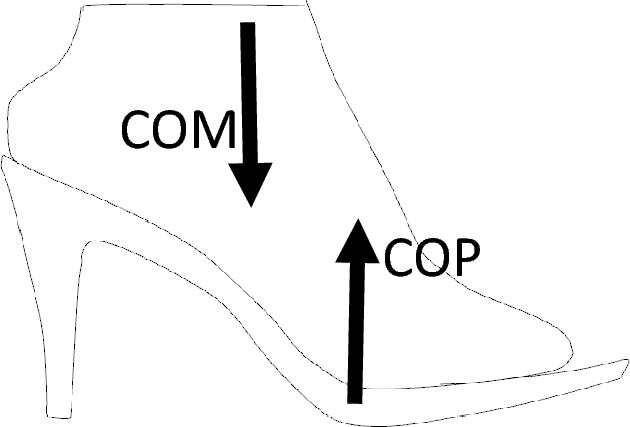
COP is anterior to COM.

Despite earlier research consistently highlighting the negative impacts of high-heeled shoes, they continue to grow in popularity among women as a fashion essential^1–4^. Contrary to popular belief, this research shows that there is a threshold time of wearing for high-heels to become painful. Beyond 3.5 h of wearing, there appears to be mild pain when considering the whole body. The reasons may be related to the time of adaptation and muscle-fatigue related time as reported in the literature where individuals alter their center of mass (COM) in relation to the center of pressure (COP)^33,34^ through structural adjustments such as trunk and pelvis rotations^10,35,36^ to modifications in muscle–tendon architecture^36–38^. According to the data, mild pain starts in the back after about 2 h followed by pain in the calcaneus, metatarsals and the toes (Fig. 4). Beyond 3.5 h, there is a rapid increase in pain in almost every part of the body part. This hypothesis needs further investigation. But, the key point here is the alteration of the COM with respect to COP. Instead, if COP is designed to match COM then the shoe would be a good design, and pain and discomfort can be reduced, if not eliminated. Figure 7 shows potential variations of the mismatch between COM and COP. If the COP is anterior to COM, then there will be an anti-clockwise moment that has to be counteracted through shoulder movement or a clockwise rotation of the pelvis increasing the lumbar lordosis. If the COP is posterior to the COM, then the opposite will happen and there will be a decrease in lumbar lordosis. This explanation agrees with the published literature where some have shown an increased lumbar lordosis^40–42^, some others decreased lordosis and kyphosis^43^, and the rest have shown no significant impact on lumbar lordosis^44,45^. After about 3.5 h of wearing, there seems to be some adaptation to shift the COM to the back of the foot to relieve the pain on the toes as there is sharp increase in the level of pain in the calcaneus and the metatarsals whereas there is only a slow increase of pain in the toes (Fig. 4). This adaptation seems to rotate the pelvis and thus we see an increase in pain in the leg and buttocks. High-heels will have no impact on lumbar lordosis when the COM variations are small and “match” the COP through appropriate footbed design by manipulating the wedge angle and shank shape^51–53^. Designing the footbed right the first time is the key to minimizing fatigue, discomfort, and pain.

Given the significant debate surrounding high-heels and their connection to pain or discomfort, it is also necessary to examine the “other factors” influencing high heels as highlighted by Baaklini et al.^43^. The heel height might not be the most crucial parameter that should be explored. It could be the shape of the footbed that influences posture, pain, discomfort, and stability because the support surface has a direct bearing on the center of pressure (COP) and the COP-COM mismatch^51^. The findings stress the importance of considering footwear characteristics to minimize discomfort and pain. Individuals should be aware of the potential consequences of wearing high heels for extended periods and choose footwear that balances style with comfort to reduce the risk of pain and discomfort.

There are several limitations in this study. Firstly, despite a total of 50 participants, the sample size within each heel height group was relatively small. Moreover, participants wore their own shoes, which were not standardized across the study, introducing some variability. Additionally, factors like BMI and foot sizes were not controlled, potentially impacting lower limb forces, particularly on different walking surfaces, and subsequently influencing foot-shoe interface pressures. However, these wide variations, inherent in the cross-sectional design, afford a more comprehensive understanding of real-life scenarios and enable the capture of diverse results, including those pertaining to time and heel height. Nonetheless, it is apparent that larger studies, incorporating greater control over potential variations of various factors, are warranted to address the aforementioned issues effectively.

## Conclusions

The study data and analysis reveal compelling trends concerning high-heeled shoe wear. Even in the most uncomfortable shoes, participants can wear them for about 3.5 h, after which pain levels consistently increase. The body areas most affected are the back, metatarsals, and the calcaneus. With “other” factors constant, an increase of 1.3 cm in heel height is estimated to correspond to a 1-point increase in back pain on a 1–10 scale. Similarly, for every additional 2-h increment of high-heeled shoe wear, back pain tends to increase by approximately 1-point. Additionally, it is observed that a heel height of up to 7.5 cm imposes a tolerable level of discomfort or pain compared to heights exceeding 7.5 cm. Discrepancies between the Center of Mass (COM) and Center of Pressure (COP) may be the primary contributors to explain the variations in the existing literature. While the COM can fluctuate with bodily adaptations, the COP can be altered through footbed design. Further research is necessary to test the proposed hypotheses to bring cohesion to the existing body of literature.

### Supplementary Information

Supplementary Information.

## Data Availability

The dataset used and analysed during the current study is available from the corresponding author on reasonable request.
