# Supplementary material for: Transient pain and discomfort when wearing high-heeled shoes
Source: Sci Rep. 2024 Apr 23;14:9291. doi: 10.1038/s41598-024-59966-9 (PMC11039660; doi:10.1038/s41598-024-59966-9)
Supplement: Supplementary file 1 — Supplementary Information. [file 41598_2024_59966_MOESM1_ESM.docx]

**Appendix A: Pilot Testing**


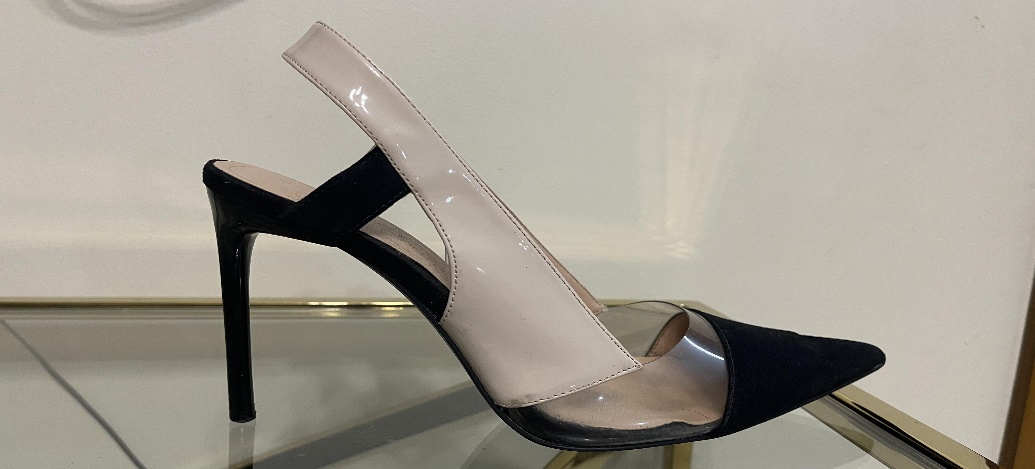


**Description:**

- Brand: Aa, size 40, 359AED, black leather Toebox, plastic covering the metatarsals, lining covering the sides of metatarsals, sling back around calcaneus. Lined with sock insole. Stiletto 11.2 cm high heel.

**Results after wearing the shoes for five hours.**

Comfortable in the first hour. In the second hour, the shoes began to bother the toes, Stiff and contracted toes.

Burning feeling in the toes (Pain level 4) and pain in metatarsals (pain level 8).

| 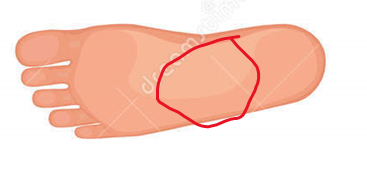 | 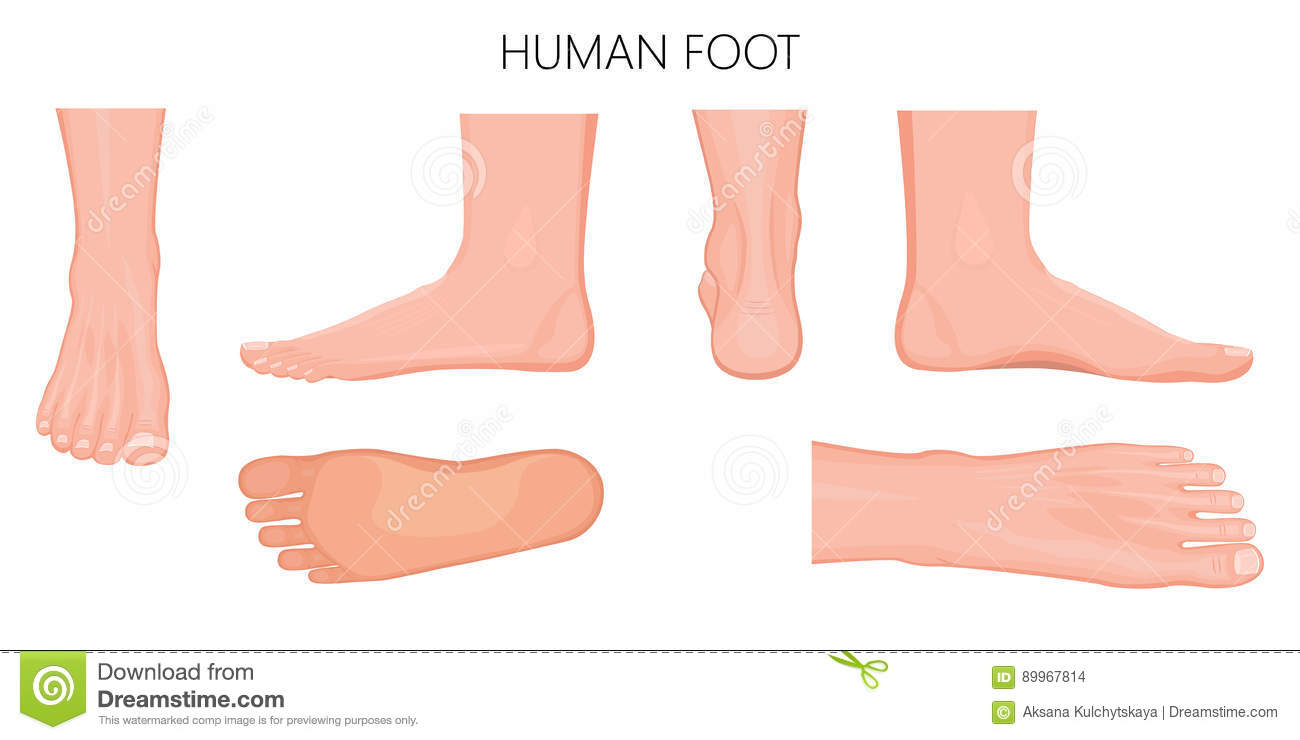 |  |
| --- | --- | --- |
| 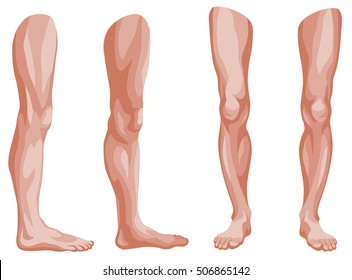  | In the third hour, a feeling of strain on the knee (pain level 3). The calf muscles were contracted for a long time (pain level 5) affecting body balance, which led to stumbling and potentially spraining the ankle (pain level 6). | 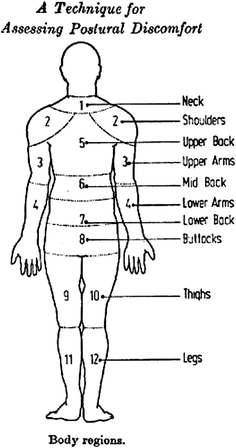 |
| 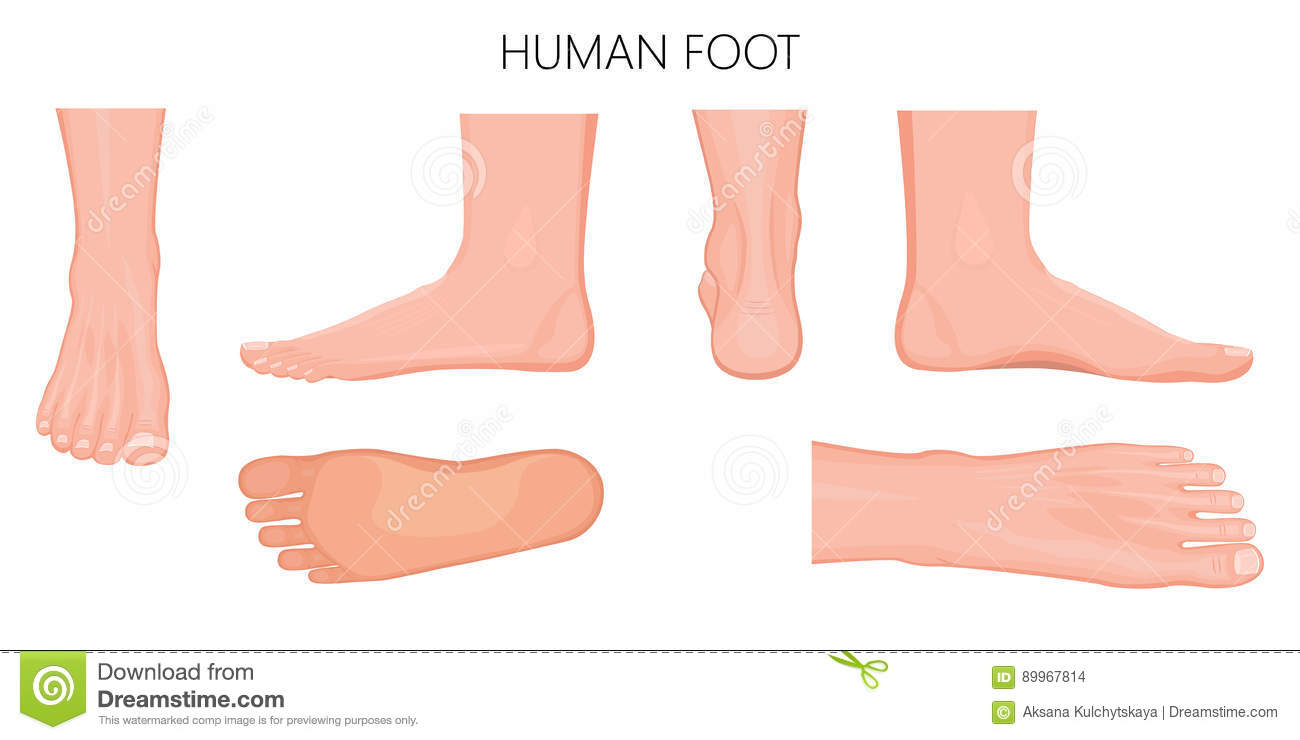  | The toenails bent, with nail going into the skin, causing severe pain (Pain level 8);  The appearance of blisters and a burning feeling in the toes, in addition to the swelling of the hallux (Pain level 7). |  |
| 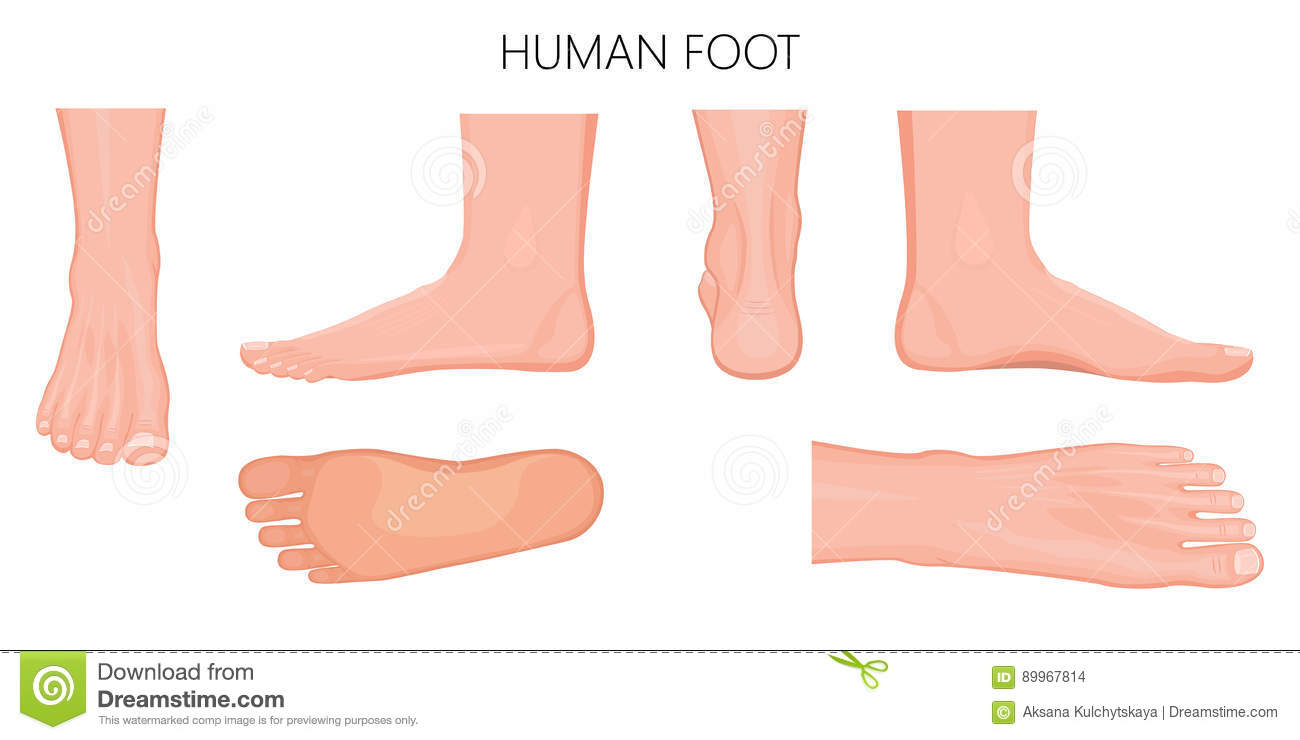 | Bruising under the nails caused redness (pain level 6). |  |
| Unable to walk due to pain after five hours (pain level 8) | | |


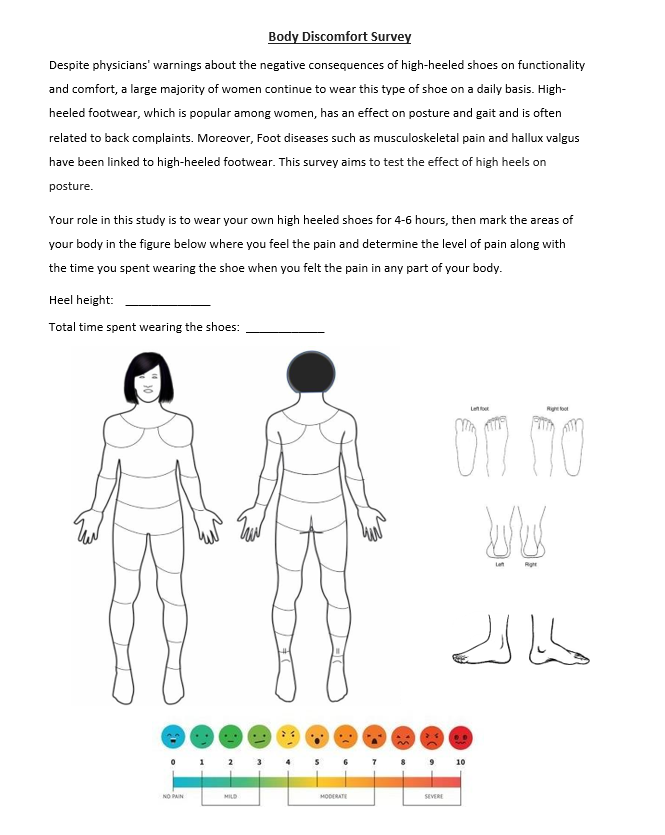
**Appendix B:**

3h/6P

3h/6P

**Appendix C**

**Sample** **survey**

h: Hours worn

P: level
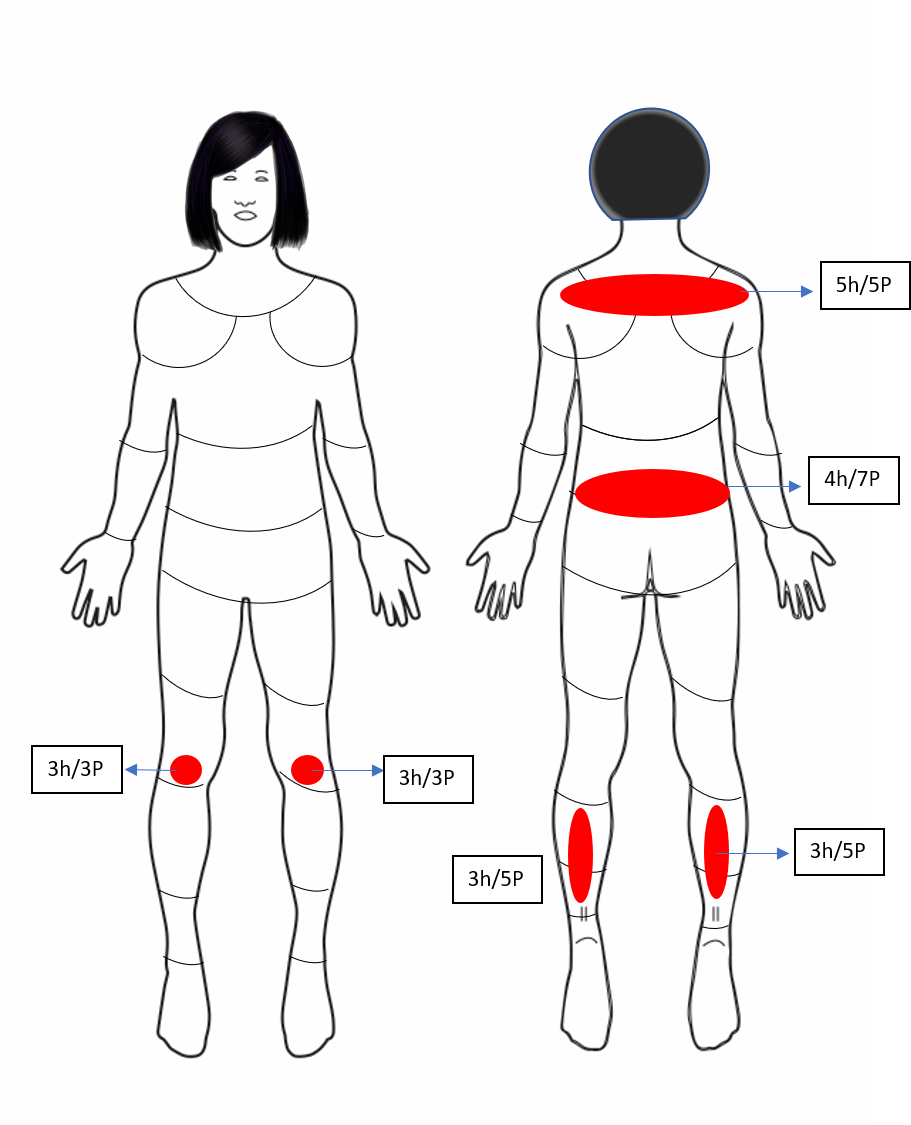
 of Pain

5h/6P

**
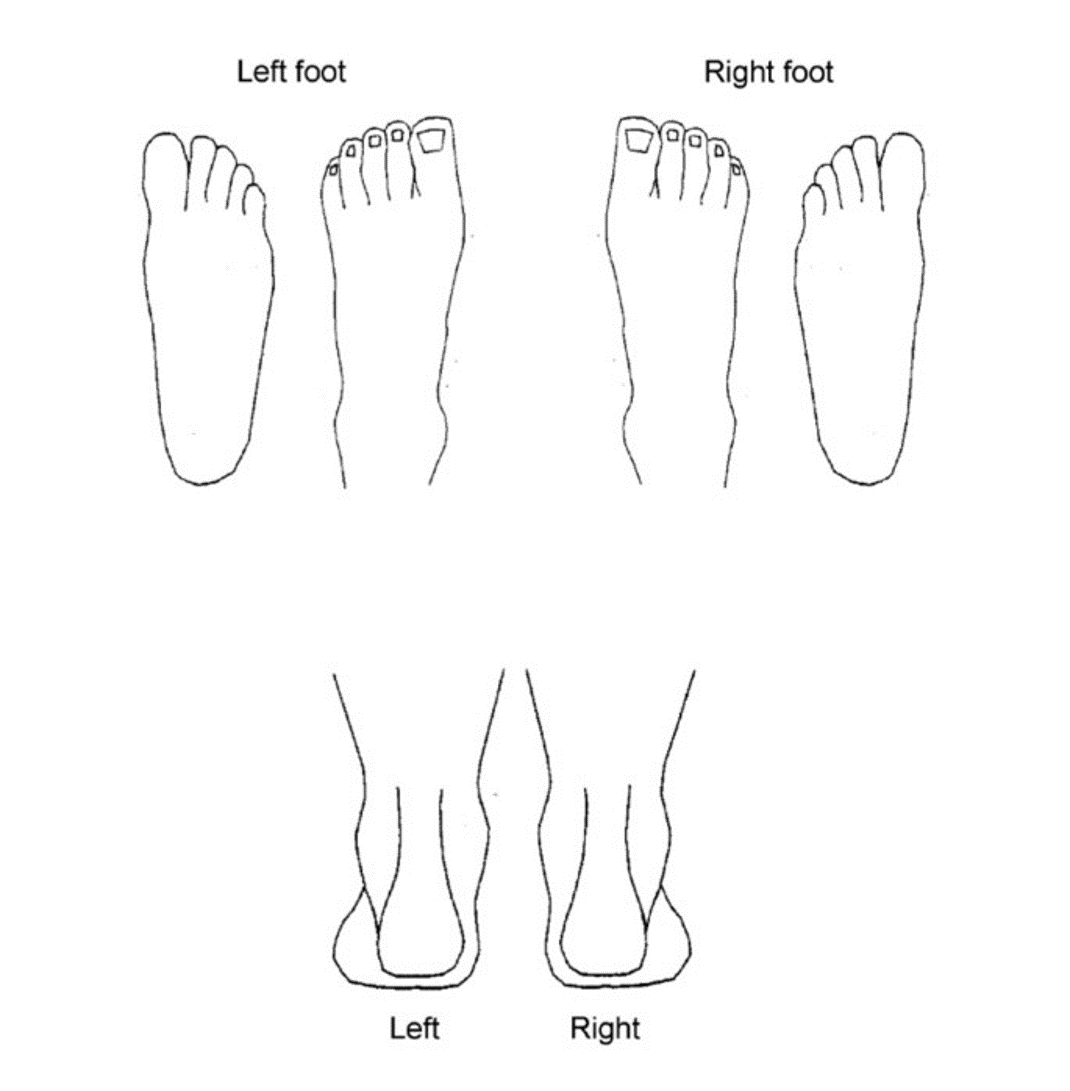
**

2h/6P

5h/7P

5h/7P

2h/6P
